# Cardiovascular Outcomes Associated With Adult Electronic Cigarette Use

**DOI:** 10.7759/cureus.9618

**Published:** 2020-08-08

**Authors:** Mohinder R Vindhyal, Hayrettin Okut, Elizabeth Ablah, Paul M Ndunda, K. James Kallail, Won S Choi

**Affiliations:** 1 Internal Medicine, University of Kansas School of Medicine-Wichita, Wichita, USA; 2 Population Health, University of Kansas School of Medicine-Wichita, Wichita, USA; 3 Population Health, University of Kansas School of Medicine-Kansas City, Kansas City, USA

**Keywords:** myocardial infarction type 1, electronic cigarette, stroke

## Abstract

Electronic cigarettes (E-Cigs) have been advertised as a safer alternative to smoking. However, E-Cigs use, like smoking, delivers ultra-small aerosol particles, which may be associated with cardiovascular disease. This study aimed to look into the association between E-Cigs use and cardiovascular disease outcomes. The study involved self-reported data from 16,855 participants from the National Health Interview Survey data from years 2014, 2016, 2017, and 2018. Results from the logistic regression analysis report E-Cigs users had higher odds of having myocardial infarction (OR 4.09, 95% CI [1.29, 12.98], P<0.05) when compared to non-users. Dual users had higher odds of myocardial infarction (OR 5.44, 95% CI [2.90, 10.22], P<0.05), stroke (OR 2.32, 95% CI [1.44, 3.74], P<0.05), and coronary artery disease (OR 2.27, 95% CI [1.37, 2.44], P<0.05) when compared to non-users.

## Introduction

Electronic cigarettes (E-Cigs) have become more popular among current cigarette smokers due to their perceived safety and targeted marketing as a potential cessation aid to regular smoking. The long-term health effects of E-Cigs use are not known, and the evidence for E-Cigs as a smoking cessation aid has also been inconclusive.

Recent prevalence data revealed that the adult users aged 25-44 years and aged 45-64 years had increased to 4.2% among adults aged 25-44 years and 2.1% among adults aged 45-64 years as per the National Health Interview Survey (NHIS) data in 2018 [1]. In the United States, the proportion of current E-Cigs users has increased to 4.8% of the adult population, and 2.3% of whom also use tobacco use (dual users), and 17.8% of whom are former users [2]. This study sought to assess and compare cardiovascular outcomes between E-Cigs users and tobacco smokers through a self-reported NHIS data questionnaire.

## Materials and methods

Participants

Data were collected from the NHIS Data Registry from 2014, 2016, 2017, and 2018 [3]. Participants were included if they answered items about their use of tobacco and E-Cigs. Respondents who reported having ever been told by a doctor or any other health professional that they had a specific condition (e.g., heart attack, stroke) was determined as having the condition. The primary outcomes were myocardial infarction, stroke, and coronary heart disease.

Measures and procedures

Once the data were downloaded securely on to a university data server, multiple variables were decoded. Variables specifically related to cardiovascular disease status were delineated along with tobacco smokers, E-Cigs users, dual users, and former smoker’s user groups for the years 2014, 2016, 2017, and 2018.

To be included in the analyses, participants had to respond to two questions: (1) if they now smoke cigarettes every day, some days, or not at all, and (2) if they currently use e-cigarettes every day, some days, or not at all. From the tobacco smoker's question, respondents could differentiate between being a “former smoker” or a “never smoker/no use.” From these two questions, participants were stratified into six groups: (1) tobacco user, (2) e-cigarette user, (3) dual user, (4) former cigarette user, and (5) former cigarette, current e-cigarette user, and (6) no user. A participant was identified as an “e-cigarette user” if she/he reported “every day” or “some days” using e-cigarettes and never using tobacco. If a participant reported being current “every day” or “some days” used tobacco and reported never using e-cigarettes, she/he was identified as a “tobacco user.” A “dual user” reported using e-cigarettes “every day” or “some days” and using cigarettes “every day” or “some days.” A “former cigarette user” reported formerly smoking and reported not currently using e-cigarettes, whereas a “former cigarette, current e-cigarette user” reported formerly smoking cigarettes and currently using e-cigarettes. If a participant reported never using tobacco or e-cigarettes, she/he was identified as a “no user.” The risk of each of the first five groups was compared to the risk of the “no user” group for the cardiovascular outcomes of myocardial infarction, stroke, and coronary heart disease.

Demographic data such as age, sex, race, ethnicity, and body mass index (BMI) were obtained. Respondents who reported having ever been told by a doctor or any other health professional that they had a specific condition (e.g., a heart attack or myocardial infarction) were identified as having the condition. The outcomes measured are myocardial infarction, stroke, and coronary artery disease.

Statistical analysis

The statistical analyses were performed using SAS 9.4 software (SAS Institute Inc., Carry, NC) and were weighted by primary sampling unit (PSU), sampling weight and, sampling stratum, as per the National Center for Health Statistics (NCHS) recommendations [4]. Logistic regression analyses with Fisher’s scoring iterative algorithm determined the covariate-adjusted odds ratios of cardiovascular outcomes for variables. Age, sex, and BMI were covariates in the multiple logistic regression models.

## Results

A total of 16,855 participants completed the survey and met the inclusion criteria. E-Cigs users were mostly male (63.3%), followed by former tobacco users, current E-Cigs users (56.5%), former tobacco users (52.9%), dual users (50.9%), and tobacco users (49.7%) (Table 1). 

**Table 1:** Participant demographics BMI, body mass index; E-Cigs, electronic cigarettes

	Percent	Frequency	Average Age (years)	Average BMI	% Male
No Tobacco Use	16.9%	2,848	30.3	27.5	56.9%
Tobacco Use	43.26%	7,291	44.0	27.9	49.7%
E-Cigs Use	2.38%	401	26.7	27.2	63.3%
Dual Use	13.28%	2,240	43.2	27.7	50.9%
Former Tobacco Use	17.42%	2,936	42.4	28.9	52.9%
Former Tobacco, Current E-Cigarette Use	6.76%	1,139	43.3	28.3	56.5%
Total	100%	16,855			

**Figure 1:**
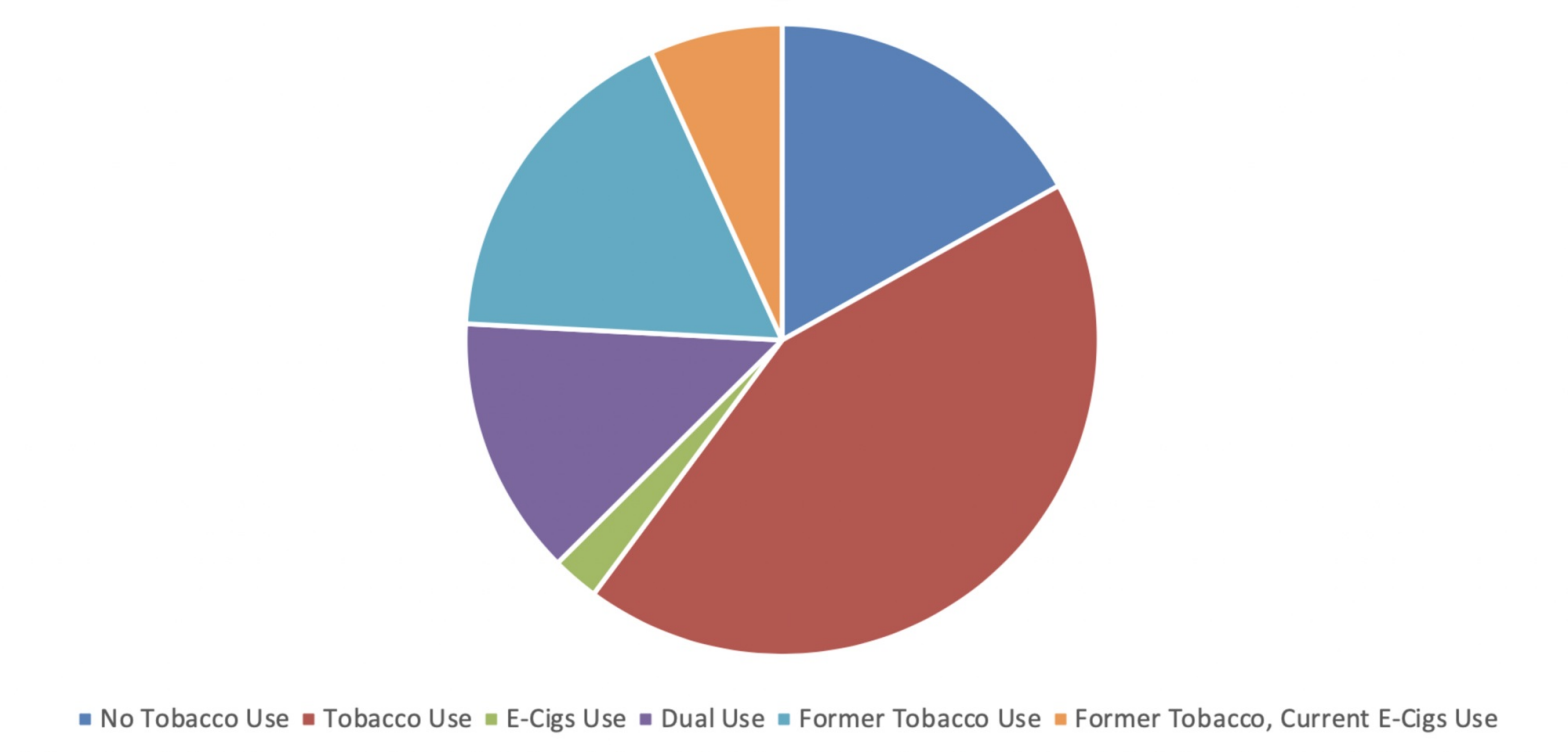
Pie chart showing different types and percentage of tobacco users

Most of the participants in this analysis were tobacco users (43.26%) followed by former tobacco users (17.42%), no tobacco users (16.9%), former tobacco users, current E-Cigs users (6.76%), and E-Cigs users (2.38%) (Figure 1). 

**Figure 2:**
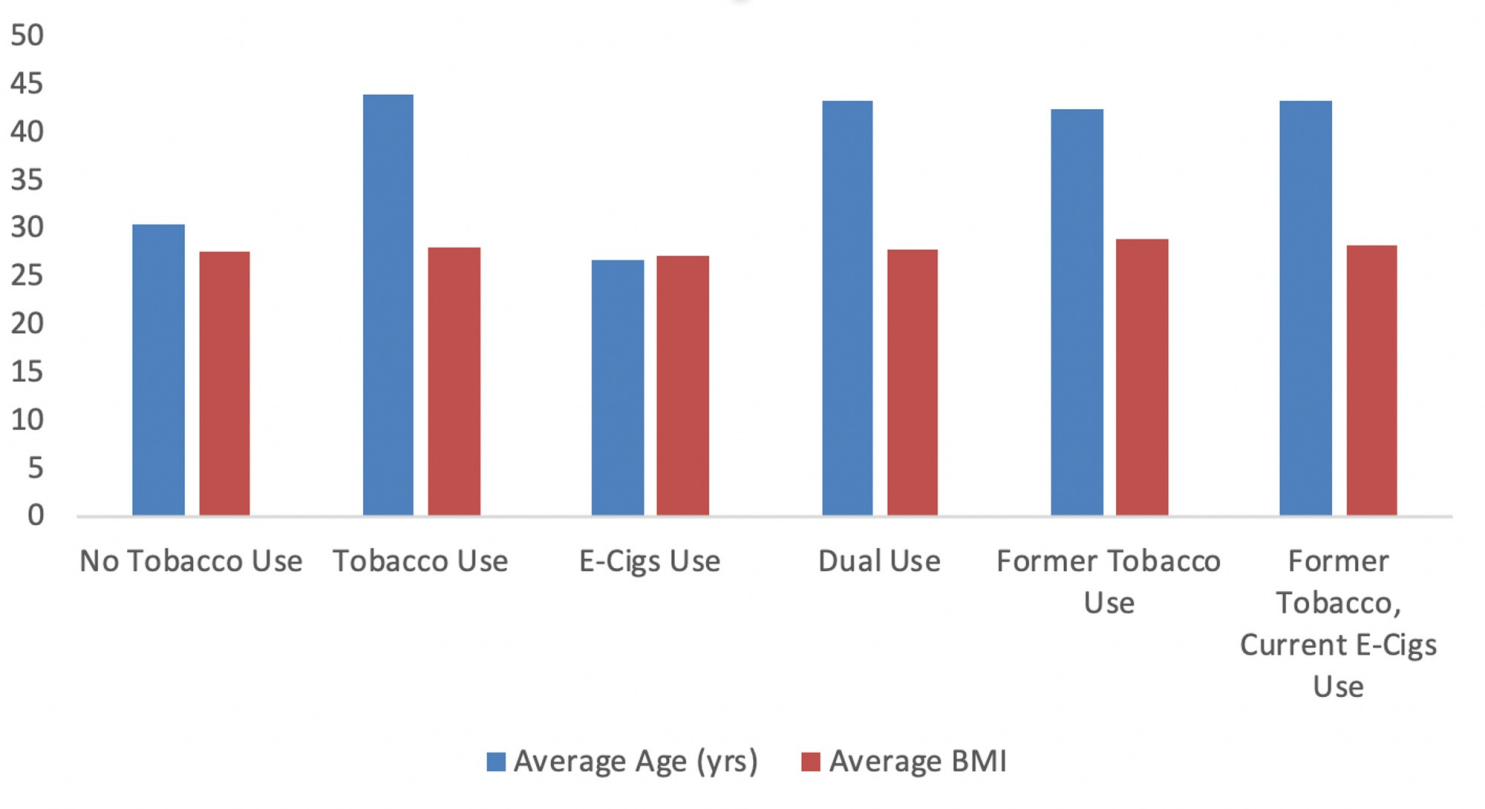
Average age (years) and body mass index (BMI) among different tobacco users

 E-Cigs users were much younger (mean age 26.7 years) compared to tobacco users (44 years) or other categories of tobacco use. The average BMI was very similar in all the user groups ranging from 27.2 to 28.9 Kg/m^2^ (Figure 2).

Results from the logistic regression analysis are presented in Table 2. All results use the “no tobacco use” category as the reference group. Dual users (tobacco and E-Cigs users) had the greatest odds of myocardial infarction (OR=5.44), followed by users of tobacco use only (OR=4.52) and current E-Cigs users (OR=4.09).

**Table 2:** Cardiovascular outcomes associated with various forms of use compared tonouse. Odds ratios (95% CIs) from logistic regression, adjusting for age sex, and body mass index (*P<0.05)

	Myocardial Infarction	Stroke	Coronary Heart Disease
No Tobacco Use	1.0	1.0	1.0
Tobacco Use	4.52 (2.49-8.21)*	2.15 (1.38-3.35)*	1.93 (1.20-3.11)*
E-Cigs Use	4.09 (1.29-12.98)*	1.22 (0.36-4.18)	0.67 (0.18-2.44)
Dual Use	5.44 (2.90-10.22)*	2.32 (1.44-3.74)*	2.27 (1.37-3.77)*
Former Tobacco Use	3.27 (1.73-6.06)*	1.73 (1.07-2.80)*	2.27 (1.39-3.71)*
Former Tobacco Use, Current E-Cigs Use	3.71 (1.89-7.28)*	1.92 (1.12-3.29)*	2.40 (1.39-4.13)*

**Figure 3:**
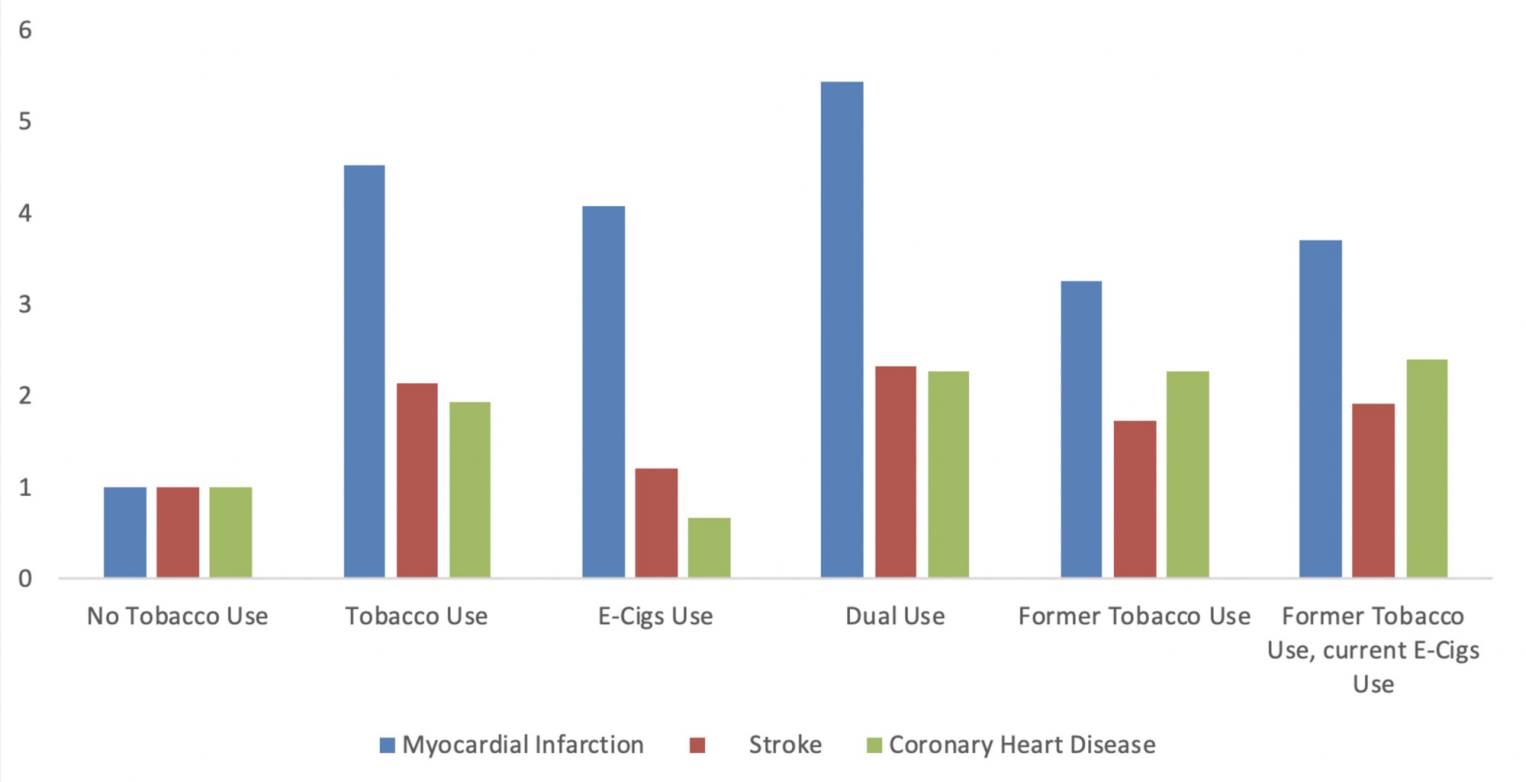
Odds of myocardial infarction, stroke, and coronary heart disease among different tobacco users.

Former tobacco users had the lowest odds of myocardial infarction compared to non-users (OR=3.27). The pattern was similar for stroke; dual users had the highest odds of stroke (OR=2.32) and former tobacco use had the lowest odds (OR=1.73). The odds of coronary heart disease were similar across most of the tobacco use categories (Figure 3).

## Discussion

The findings from this study confirm prior studies that E-Cigs use is associated with increased odds of myocardial infarction, stroke, and coronary heart disease [5,6]. Those who reported being dual users appear to be the most likely associated with myocardial infarction, stroke, or coronary heart disease. These results indicate that the dual use of both tobacco use and E-Cigs is more dangerous than the use of either tobacco products alone for myocardial infarction or stroke.

NHIS is a cross-sectional study; therefore, it cannot truly determine cause and effect, but it can determine the association at the time of the study. Different types and designs of E-Cigs and how they are used will yield different exposures. Data were not available to evaluate exposure dose in any meaningful way (i.e., duration of use, frequency of use, and type of product used), which is critical to assess whether using E-Cigs exposure is a risk for cardiovascular disease. The NHIS study data are self-reported which is subject to the recall bias, and one also has to account for social desirability where the individuals in the survey may answer questions less honestly in a way which are socially acceptable and desirable [7]. Further, when one is reviewing self-reported data, subject fatigue, memory burden, and confusion when answering the questions must be considered. There is always a discrepancy among participants’ responses and clinical validation of the diagnosis, as reported from previous studies [8,9]. The definition of E-Cigs usage and the amount and duration of its usage have not been detailed enough, which may indicate that the data for E-Cigs users may not be accurate when accounted for several risk factors.

Another drawback of the study is that the participants may already have had cardiovascular morbidity such as myocardial infarction, coronary artery disease, and stroke before using E-Cigs. Factors such as family history and medication usage, which may play a role in cardiovascular disease, were not documented from the questionnaire. This study, along with other cross-sectional E-Cigs studies, may not infer causality, but this is one among the first studies that suggest there are harmful cardiovascular outcomes associated with E-Cigs use, especially when paired with the use of tobacco.

## Conclusions

E-Cigs use, when compared with non-users, is associated with increased risk of myocardial infarction. Similarly, former tobacco users and former tobacco users, current e-cigarette users have increased risk for myocardial infarction, stroke, and coronary heart disease. The highest risk for myocardial infarction, stroke, and coronary heart disease was seen with dual users. 

Our study established an association between negative health outcomes and e-cigarette use and tobacco use. A need remains for more longitudinal cohort studies to establish the causation linkage for the cardiovascular outcomes and e-cigarette usage.
